# Heritability of hoarding symptoms across adolescence and young adulthood: A longitudinal twin study

**DOI:** 10.1371/journal.pone.0179541

**Published:** 2017-06-28

**Authors:** Volen Z. Ivanov, Ashley Nordsletten, David Mataix-Cols, Eva Serlachius, Paul Lichtenstein, Sebastian Lundström, Patrik K. E. Magnusson, Ralf Kuja-Halkola, Christian Rück

**Affiliations:** 1 Centre for Psychiatry Research, Department of Clinical Neuroscience, Karolinska Institutet, Stockholm, Sweden; 2 Stockholm Health Care Services, Stockholm County Council, Stockholm, Sweden; 3 Department of Medical Epidemiology and Biostatistics, Karolinska Institutet, Stockholm, Sweden; 4 Centre for Ethics Law and Mental Health, University of Gothenburg, Gothenburg, Sweden; 5 Gillberg Neuropsychiatry Centre, Institution of Neuroscience and Physiology, University of Gothenburg, Gothenburg, Sweden; Universitair Medisch Centrum Groningen, NETHERLANDS

## Abstract

**Background:**

Twin studies of hoarding symptoms indicate low to moderate heritability during adolescence and considerably higher heritability in older samples, suggesting dynamic developmental etiological effects. The aim of the current study was to estimate the relative contribution of additive genetic and environmental effects to hoarding symptoms during adolescence and young adulthood and to estimate the sources of stability and change of hoarding symptoms during adolescence.

**Methods:**

Univariate model-fitting was conducted in three cohorts of twins aged 15 (n = 7,905), 18 (n = 2,495) and 20–28 (n = 6,218). Longitudinal analyses were conducted in a subsample of twins for which data on hoarding symptoms was available at both age 15 and 18 (n = 1,701).

**Results:**

Heritability estimates for hoarding symptoms at ages 15, 18 and 20–28 were 41% (95% confidence interval [CI]: 36–45%), 31% (95% CI: 22–39%) and 29% (95% CI: 24–34%) respectively. Quantitative sex-differences emerged in twins aged 15 at which point the heritability in boys was 33% (95% CI: 22–41%) and 17% (95% CI: 0–36%) in girls. Shared environmental effects played a negligible role across all samples with the exception of girls aged 15 where they accounted for a significant proportion of the variance (22%; 95% CI 6–36%). The longitudinal bivariate analyses revealed a significant phenotypic correlation of hoarding symptoms between ages 15 and 18 (0.40; 95% CI: 0.36–0.44) and a strong but imperfect genetic correlation (0.75; 95% CI: 0.57–0.94). The bivariate heritability was estimated to 65% (95% CI: 50–79%).

**Conclusions:**

Hoarding symptoms are heritable from adolescence throughout young adulthood, although heritability appears to slightly decrease over time. Shared environmental effects contribute to hoarding symptoms only in girls at age 15. The stability of hoarding symptoms between ages 15 and 18 is largely explained by genetic factors, while non-shared environmental factors primarily have a time-specific effect. The findings indicate that dynamic developmental etiological effects may be operating across the life span.

## Introduction

Hoarding disorder (HD) is characterized by a profound inability to discard possessions, resulting in the obstructive and hazardous accumulation of clutter throughout the sufferer’s living environment and significant distress and impairment [1]. Most individuals with HD also excessively acquire posessions that they do not need or have space for. Insight ranges from good to delusional [2, 3].

While HD typically comes to the attention of clinical services late in life when the home has become severely cluttered, retrospective patient reports have consistently suggested that the early manifestations of hoarding symptoms can be traced early in life. The symptoms are then thought to follow a deteriorating course over the lifespan [4–6]. Recent population-based work [7–9] has offered empirical support for these early retrospective reports, with results indicating that at least 1% of adolescents may endorse clinically-significant hoarding symptoms, a somewhat lower point prevalence than in estimates derived among adult samples [10–12]. Subsequently, from age 15, the prevalence of clinically significant hoarding symptoms is suggested to increase linearly with around 20% every 5 years [9]. Whether hoarding symptoms are distributed equally between the sexes during adolescence remains an unresolved issue, with some studies suggesting a higher prevalence in girls [7, 8, 13] while others have not detected any sex-differences [14].

Although, the etiology of hoarding symptoms is still largely unknown, evidence from twin studies indicates that, in adults, genetic factors account for a substantial proportion of the phenotypic variance, with heritability estimates ranging from 0.36 to 0.49 [10, 15, 16]. However, our prior work [7] suggests that hoarding symptoms may be less heritable during adolescence. Specifically, in a study of 3,974 twins aged 15, genetic factors explained 32% of the variance in boys and only 2% in girls [7]. Potential etiological sex-differences should however, be interpreted cautiosly since other more recent studies of young twins from The Netherlands Twin Register [15, 17] did not detect such sex-differences. Taken together, the findings of twin studies from different timepoints across the lifespan, suggest the possibility of dynamic changes in the etiology of hoarding symptoms, with the influence of genetics and environment varying over time and, potentially, between the sexes. Previous heritability estimates of hoarding symptoms have, however, entirely relied on single-timpeoint, cross-sectional methods–a design which does not offer scope for evaluating time-change in symptoms or, indeed, the genetic and environmental influences underlying such change. To map the dynamic etiology suggested by current resesearch, alternative approaches are therefore required. Given the mounting evidence implicating the adolescent period in the emergence and establishment of hoarding difficulties, and established knowledge of the progressive burden incurred by these symptoms from onset through the lifecourse, investment in such approaches appears warranted. Longitudinal twin studies of obsessive-compulsive symptoms (OCS), a related phenotype, have indicated that these symptoms are moderately stable across childhood and adolescence, and that such stability is largely, but not entirely, explained by genetic factors [18, 19]. These findings have been interpreted as representing developmentally dynamic processes in the genesis of OCS [18].

The primary objective of the current study was to asssess the relative contributions of genetic and environmental factors (both shared and nonshared) to hoarding symptoms in three large cohorts of young twins aged 15, 18 and 20–28. Based on the previous literature, we hypothesized that heritability would increase, and the impact of shared and non-shared enviromental influences decrease, with increasing age. A second objective was to estimate the sources of stability and change of hoarding symptoms during adolescence by performing longitudinal bivariate twin analyses on a subsample of twins who had data on hoarding symptoms at both ages 15 and 18. Based on the previous OCS literature, we predicted that hoarding symptoms would be moderately stable across adolescence and that genetic factors would explain a substantial, but incomplete, proportion of that stability.

## Materials and methods

### Sample

Participants were monozygotic (MZ) and dizygotic (DZ) twins enrolled in the population-based Swedish Twin Registry who took part in one of two large cohort studies; (1) the Child and Adolescent Twin Study in Sweden (CATSS) [20] and (2) adult twins, aged 20–28, from the Young Adult Twins in Sweden Study (YATSS 20–28). CATSS is a prospective, longitudinal study of all twins born in Sweden since 1992. Parents of these twins were initially contacted and interviewed when the twins were 9 or 12 years old. In the current analysis, we have used information on hoarding symptoms collected from the follow-ups at ages 15 (CATSS-15) and 18 (CATSS-18). At these time points, the twins were contacted directly and asked to complete a questionnaire battery on several neurodevelopmental childhood-onset disorders, including a measure of hoarding symptoms.

In CATSS-15, hoarding data were available for 7,905 twin individuals (response rate = 51% of all Swedish twins in the age group). 3,974 of these individuals participated in our previous study (of which, 3,110 with known zygosity were included in the twin analyses) [7]. The CATSS-18 sample included 2,495 individuals with available hoarding data (response rate = 48%). Additionally, a subset of twins (n = 1,701) for which data at both age 15 and 18 was available, were included in the longitudinal analysis.

The YATSS 20–28 survey ran from March 2013 to January 2014 and was sent out to twin pairs who had not been previously contacted by the Swedish Twin Registry. This target sample included nearly all young adult twins born in Sweden from May 1985 to June 1992. Twins (n = 1,001) who had declined further participation in the twin registry, had protected identity, had migrated, or had died were not contacted. This survey was comprised of items evaluating obsessive-compulsive and related symptoms (including hoarding), gastrointestinal diseases, lung diseases, arthritis, asthma, allergies, affective disorders, eating disorders, fibromyalgia, chronic fatigue syndrome and women’s health. Data on hoarding symptoms were available for 6,218 individual twins (response rate = 38%). Twins in YATSS 20–28 were initially contacted with a letter containing information about the study, a personal identification code and a password with which they could log on to the survey website. Twins who did not respond to the survey received one reminder via telephone. To ensure that twins who did not have Internet access could partake in the survey, the option of participating in a telephone version of the survey was also offered. Twins who participated received either a cinema ticket or a gift voucher worth 120 SEK (15 USD) and took part in a competition to win a tablet computer.

Twin zygosity across all samples was established by a test based on a panel of 47 thoroughly validated single nucleotide polymorphisms (SNPs) [21]. Zygosity was calculated as the likelihood of being monozygotic versus dizygotic based on data from the genotyping. If DNA was unavailable, an algorithm based on twin similarity that correctly classifies > 95% of twins compared to DNA testing was used [22].

The Regional Ethics Review Board in Stockholm approved all data collection points and samples included in the study under contracts 02–289, 03–672, 2009–793 and 2012/2107-31/3. According to Swedish regulations and the board’s decision, we were allowed to obtain written informed consent directly from all participants who were underage in this study and instead of from their parents. Parents of all these twins were however informed about the study and what data was being collected.

### Measures

All participants in the current investigation filled out the Hoarding Rating Scale-Self Report (HRS-SR) [23]. The HRS-SR consists of five items measured on a 9-point Likert type scale ranging from 0 (none) to 8 (extreme) yielding a total score between 0 and 40. Four of the five scale items reflect the DSM-5 criteria for HD: clutter in the rooms of the home, difficulty discarding possessions, distress and impairment, while one item, excessive acquisition, is a diagnostic specifier in DSM-5. Since it is highly likely that adolescents exert limited control over their entire homes, the clutter item in the questionnaire in CATSS-15 and CATSS-18 was rephrased to refer to clutter in the young person’s own room and not in the entire home. This modification was not made to the questionnaire in YATSS 20–28, based on the assumption that most young adults have moved away from their parents’ home and have control over their living environments. However, due to a restriction in the magnitude of items in the questionnaire battery in YATSS 20–28, the impairment item was removed in the questionnaire sent out to this twin cohort. Thus, the HRS-SR in YATSS 20–28 included four items (rather than five as in the original version), with total scores ranging from 0–32 (rather than 0–40). Table 1 displays the psychometric properties of the HRS-SR in the 3 cohorts. Consistent with the literature, a principal component analysis revealed a single factor structure explaining 45.6% to 48.5% of the variance. Internal consistency (Cronbach’s α) ranged from 0.64 to 0.70.

**Table 1:** Internal consistency (Cronbach’s α) and factor loadings for the Hoarding Rating Scale-Self Report in 3 cohorts of Swedish twins aged 15, 18 and 20–28 years.

Cohort	HRS-SR item	Cronbach’s α	Factor 1 loading^a^
**CATSS-15**		0.70	
	Difficulty discarding		0.66
	Excessive acquisition		0.68
	Clutter		0.62
	Distress		0.72
	Impairment		0.69
**CATSS-18**		0.70	
	Difficulty discarding		0.65
	Excessive acquisition		0.70
	Clutter		0.62
	Distress		0.71
	Impairment		0.70
**YATSS 20–28**		0.64	
	Difficulty discarding		0.71
	Excessive acquisition		0.75
	Clutter		0.64
	Distress		0.68

**Note:** HRS-SR = Hoarding Rating Scale-Self Report.

^a^ Single factor structure derived from a principal component analysis explaining 45.6% of the variance in CATSS-15, 47.7% in CATSS-18 and 48.5% in YATSS 20–28.

In the CATSS samples, we defined presence of clinically significant hoarding symptoms as scoring at least moderate severity (4 or higher out of 8) on items measuring clutter and difficulties discarding and on at least one of the items measuring distress and impairment. This procedure has previously been described as criteria for clinically significant hoarding [24, 25] and closely resembles the DSM-5 criteria [1]. The same procedure was used in YATSS 20–28 with the impairment item omitted from the algorithm.

Due to positive skewness in total HRS-SR scores in all three twin cohorts (CATSS-15 skewness = 1.59; CATSS-18 skewness = 1.76; YATSS 20–28 skewness = 1.38), a logarithmical transformation using the natural logarithm was performed on the total scores, which resulted in reduced skewness (CATSS-15 skewness = -0.13; CATSS-18 skewness = 0.06; YATSS 20–28 skewness = -0.14).

### Statistical analyses

Basic statistical analyses were performed using the SAS software version 9.3 [26]. In the prevalence estimates, which were based on a published algorithm used to define clinically significant hoarding symptoms [24], we controlled the precision (i.e., the confidence intervals) for the clustering of twins within families. All model fitting analyses were performed with the structural equation modelling package OpenMx [27] version 2.3.1 in the R software [28]. All parameter estimates in the twin analyses were obtained from full maximum-likelihood estimates in OpenMx, which enables handling of missing data and the inclusion of singletons. Goodness of fit was examined by a likelihood ratio test chi-square statistic. Akaike’s information criterion (AIC; [29]) was computed for every model and the model with the lowest AIC considered as the model with the best fit.

### Twin analyses

In the current study, we included both univariate and bivariate (longitudinal) twin analyses. Univariate analyses included model-fitting analyses in each of the three cohorts separately. Longitudinal analyses included individuals who responded to both CATSS waves. Only twins with known zygosity were included in the model-fitting analyses (CATSS-15: n = 7,167; CATSS-18: n = 2,223; YATSS 20–28: n = 5,991). All pairs where at least one twin had available data were included in the twin analyses. All model-fitting analyses were based on HRS-SR total scores.

The twin design [15] is based on the comparison of differing genetic similarity between MZ twins, who share all of their genes, and DZ twins, who on average share 50% of their segregating genes. A stronger within-pair resemblance in MZ compared to DZ twins on a phenotype is due to a genetic influence on that trait, assuming that both twin types grow up under equal environments. Twin models aim to decompose the variance of phenotypes into additive genetic factors (A), shared environmental factors (C; which make twins within a pair alike), and non-shared environmental factors (E; which make twins within a pair dissimilar). Measurement error is also included in E. The within-variable cross twin resemblance was estimated by calculating intraclass correlations for the HRS-SR total scores in MZ and DZ twins.

First, univariate model fitting was performed for each age sample separately. In the first step, a fully saturated model was fitted for each time point to estimate the means and covariance matrices that provided the best fit for our data. In the YATSS 20–28 sample, we additionally adjusted means for age because ages in this cohort ranged between 20 and 28. To test the appropriateness of the data for twin modelling we performed a series of assumption tests; we equated the means and variances in the saturated model across twin order, zygosity and sex and investigated whether any of these restrictions produced a worse fit. Likelihood ratio tests comparing the current, nested model to the previous model with the best fit were performed.

In CATSS-18 and YATSS 20–28, the correlational patterns did not indicate any possible sex differences and thus no sex-limitation models were fitted to the data. However, as in a previous study of hoarding symptoms in a small subset of this cohort [7], the correlational pattern in CATSS-15 indicated a markedly greater difference between MZ and DZ intraclass correlations in males compared to females, suggesting sex differences in genetic and environmental influences on hoarding symptoms. We therefore fitted several sex-limitation models to the CATSS-15 data to test for both quantitative and qualitative sex differences.

Quantitative sex differences refer to sex differences in the magnitude of the effects of A, C and E on the variance of a trait. In the model fitting, this is reflected by allowing the effects of A, C and E to be estimated separately in both sexes, whereas the genetic correlation between dizygotic opposite-sex twins is fixed to 0.50. Qualitative sex differences refer to sex differences in the sets of genes acting on a trait. This is achieved by allowing the genetic correlation between opposite sex-twins to be lower than expected (0.50), indicating that different genetic sources are operating in males and females. In total, we fitted four sex-limitation models to the CATSS-15 data: 1) a full sex-limitation model, which allows both quantitative and qualitative sex differences; (2) a sex-limitation model, in which only quantitative sex-differences are allowed; (3) a sex-limitation model, where only qualitative sex differences are allowed and (4) a no sex-limitation model, in which A, C and E are all restrained to be equal on both sexes, thus not allowing any sex differences.

For the longitudinal analyses, we calculated two additional correlations: (1) a phenotypic correlation (*r*PH) of hoarding symptoms between ages 15 and 18 and (2) cross-twin-cross-time (CTCT) correlations (i.e. the correlation between hoarding symptoms at age 15 in twin 1 and at age 18 in twin 2, and vice versa) for MZ and DZ twins. Larger CTCT correlations in MZ twins compared to DZ twins provide an indication that the phenotypic correlation between two time points is at least partly explained by genetic factors.

In order to partition the covariance between hoarding symptoms at ages 15 and 18 into genetic and environmental components, we also performed longitudinal bivariate twin analyses. In bivariate longitudinal analyses, the additive genetic (*r*G), shared environmental (*r*C) and non-shared environmental (*r*E) correlations are estimated, with a value of 1.0 indicating a complete overlap across two time points in either genetic or environmental factors on the trait. In the final step of the bivariate analyses, we estimated the proportion of the phenotypic covariation between age 15 and 18 explained by shared genetic factors (bivariate heritability; Biv A), shared environmental factors (Biv C) and non-shared environmental factors, (Biv E). In the bivariate longitudinal models, we also allowed for different means in females and males.

## Results

### Sample description

All twins with complete HRS-SR scores were included in estimation of the prevalence. Mean HRS-SR scores, prevalence of clinically significant hoarding symptoms and zygosity groups in all samples are displayed in Table 2. In CATSS-15, 45% (n = 3,589) of the twins were boys and 55% (n = 4,316) girls. Similarly, in CATSS-18, 42% (n = 1,047) were boys and 58% (n = 1,448) girls. The mean age of participants in the YATSS 20–28 cohort was 23.8 years (SD = 2.0, median = 24.0, range 20–28). This sample was comprised of 39% (n = 2,448) males and 61% (n = 3,770) females.

**Table 2:** Demographic and clinical characteristics of the participants in Swedish twins at age 15, 18 and 20–28 years.

Cohort	CATSS-15	CATSS-18	YATSS 20–28
Total, *n*	7,905	2,495	6,218
MZm, *n* (%)	922 (11.66)	247 (9.9)	1,006 (16.2)
MZf, *n* (%)	1,234 (15.6)	433 (17.4)	1,633 (26.3)
DZm, *n* (%)	1,158 (14.7)	337 (13.5)	616 (9.9)
DZf, *n* (%)	1,219 (15.4)	370 (14.8)	975 (15.7)
DZos, *n* (%)	2,634 (33.3)	846 (33.9)	1,761 (28.3)
Unknown zygosity, *n* (%)	738 (9.3)	262 (10.5)	227 (3.7)
Twins from complete pairs, *n*	6,254	1,786	4,028
Singletons, *n*	913	447	1,963
Mean HRS-SR score (SD)	4.4 (4.7)	3.8 (4.5)	3.8 (3.9)^a^
Hoarding symptoms, % (95% CI)	1.5 (1.3–1.8)	0.9 (0.6–1.4)	0.8 (0.6–1.1)

**Note**: HRS-SR = Hoarding Rating Scale-Self Report; SD = standard deviation; CI = confidence interval; MZm = male monozygotic twins; MZf = female monozygotic twins; DZm = male dizygotic twins; DZf = female dizygotic twins; DZos = opposite-sex dizygotic twins.

^a^ Mean score based on 4 items (difficulty discarding, clutter, acquisition and distress) from HRS-SR.

### Twin correlations

Twin correlations for all three samples are shown in Table 3. MZ twin correlations were larger than DZ correlations in all the samples, suggesting genetic effects on hoarding symptoms. However, MZ correlations were less than 1.0, indicating probable substantial effects of non-shared environmental effects (and measurement error). In CATSS-15, we found a significant difference in intraclass correlations between the three dizygotic groups (p<0.001), indicating possible sex effects on the genetic and environmental contributions to hoarding symptoms at age 15.

**Table 3:** Intraclass correlations for the HRS-SR in Swedish twins at age 15, 18 and 20–28 years.

Zygosity (Cohort)	Males	Females	Opposite-sex	Equated	*P*
MZ (CATSS-15)	0.35 (0.26–0.43)	0.38 (0.31–0.45)	-	0.37 (0.31–0.42)	0.514
DZ (CATSS-15)	0.15 (0.06–0.23)	0.31 (0.23 0.38)	0.12 (0.06–0.18)	0.17 (0.13–0.22)	<0.001
MZ (CATSS-18)	0.15 (-0.04–0.33)	0.31 (0.18–0.43)	-	0.26 (0.15–0.36)	0.148
DZ (CATSS-18)	0.19 (0.02–0.35)	0.14 (-0.02–0.29)	0.12 (0.01–0.23)	0.14 (0.06–0.22)	0.777
MZ (YATSS 20–28)	0.33 (0.21–0.44)	0.34 (0.25–0.42)	-	0.34 (0.27–0.40)	0.878
DZ (YATSS 20–28)	0.15 (-0.05–0.33)	0.23 (0.09–0.36)	0.12 (0.00–0.23)	0.16 (0.08–0.24)	0.460

**Note:** MZ = Monozygotic twins; DZ = Dizygotic same sex twins. Equated = value obtained when correlations are assumed to be equal across groups. *P* = p-value of the likelihood ratio test when comparing a model allowing separate correlations in gender groups to model where they were restricted to be equal across groups.

### Univariate model fitting

Table 4 provides parameter estimates from the best fitting models across samples. The assumption testing did not show any differences in means and variances across twin order, zygosity and sex (all p > 0.05). Results showed that, in all three samples, the shared environmental parameter could be dropped without a significant result in model fit. Dropping the additive genetics parameter (A) however, resulted in reduced fit. Thus, the best fitting model across all samples was the AE model (see Table 5 for model-fitting results). The proportion of the variance accounted for by genetic effects was 41% (95% CI: 36–0.45%) in CATSS-15, 31% (95% CI: 22–39%) in CATSS-18 and 29% (95% CI: 24–34%) in YATSS 20–28. The contribution of non-shared environmental effects to the variance was 59% (95% CI: 55–64%) in CATSS-15, 69% (95% CI: 62–78%) in CATSS-18 and 71% (95% CI: 66–76%) in YATSS 20–28.

**Table 4:** Explained variance by additive genetic and environmental factors to hoarding symptoms in Swedish twins at age 15, 18 and 20–28 years according to best fitting model.

			Sex-limitation					
	Total sample		Males			Females		
Cohort	A	E	A	C	E	A	C	E
**CATSS-15**	0.41 (0.36–0.45)	0.59 (0.55–0.64)	0.33 (0.22–0.41)	0.01 (0.00–0.08)	0.66 (0.58–0.74)	0.17 (0.00–0.36)	0.22 (0.06–0.36)	0.61 (0.55–0.68)
**CATSS-18**	0.31 (0.22–0.39)	0.69 (0.62–0.78)	-	-	-	-	-	-
**YATSS 20–28**	0.29 (0.24–0.34)	0.71 (0.66–0.76)	-	-	-	-	-	-

**Note:** A = additive genetic effects; C = shared environmental effects; E = non-shared environmental effects and measurement error.

**Table 5:** Model-fitting results for hoarding symptoms in Swedish twins at age 15, 18 and 20–28 years.

Cohort	Model name	-2ll	Estimated parameters	AIC
**CATSS-15**	Saturated	19271.02	10	4953.020
	ACE	19275.71	4	4945.714
	**AE**	**19275.71**	**3**	**4943.714**
	CE	19317.06	3	4985.061
	E	19540.83	2	5206.831
**CATSS-15 Sex-limitation**	Full sex-limitation^a^	17777.20	9	4277.202
	**Quantitative sex-limitation**^**b**^	**17777.20**	**8**	**4275.202**
	Qualitative sex-limitation^c^	17784.69	6	4278.690
	No sex-limitation^d^	17786.36	5	4278.365
**CATSS-18**	Saturated	6043.767	10	1597.767
	ACE	6051.539	4	1593.539
	**AE**	**6051.629**	**3**	**1591.629**
	CE	6055.581	3	1595.581
	E	6093.648	2	1631.648
**YATSS 20–28**	Saturated	9165.883	10	2087.883
	ACE	9171.010	4	2081.010
	**AE**	**9171.010**	**3**	**2079.010**
	CE	9182.442	3	2090.442
	E	9263.424	2	2169.424
**Longitudinal**	Bivariate ACE	24617.76	13	5839.756
	**Bivariate AE**	**24618.80**	**10**	**5834.804**
	Bivariate CE	24644.57	10	5860.569
	Bivariate E	24885.81	7	6095.805

**Note:** -2LL = minus twice the log likelihood; AIC = Akaike’s Information Criterion; A = additive genetic effects; C = shared environmental effects; E = non-shared environmental effects and measurement error.

The best fitting model is bolded.

^a^ The full sex-limitation model allows both quantitative and qualitative sex differences.

^b^ The quantitative sex-limitation model allows only quantitative sex-differences.

^c^ The qualitative sex-limitation model allows only qualitative sex-differences.

^d^ The no sex-limitation model does not allow any sex-differences.

Since the correlational pattern in CATSS-15 indicated possible sex-differences, these were further investigated by fitting sex-limitation models to the data from this sample. The model-fitting results indicated that the model with the lowest AIC was the quantitative effects sex-limitation model, suggesting sex-differences in the magnitude, but not sources, of genetic effects. According to this model, in boys in CATSS-15, genetic factors explained 33% (95% CI: 0.22–0.41) while shared environmental factors accounted for a negligible 1% (95% CI: 0.00–0.08) of the variance. The remaining variance, 66% (95% CI: 0.58–0.74), was explained by non-shared environmental factors. In girls, additive genetic factors explained 17% (95% CI: 0.00–0.36) of the variance. In contrast to boys, in girls, shared environmental factors had a significant effect on the variance of hoarding symptoms: 22% (95% CI: 0.06–0.36). Finally, as in boys, non-shared environmental factors strongly influenced hoarding symptoms at age 15 in girls: 61% (95% CI: 0.55–0.68).

### Longitudinal analyses

Results of the bivariate longitudinal twin analyses are displayed in Table 6. We found a moderate phenotypic correlation (*r*Ph: 0.40 [95% CI: 0.36–0.44]) between hoarding symptoms at age 15 and 18 in the same individuals. Furthermore, inspection of CTCT correlations showed that MZ correlations (r = 0.25 [95% CI: 0.18–0.32]) were higher compared to DZ correlations (r = 0.15 [95% CI: 0.09–0.20]), indicating possible genetic effects on the covariance of hoarding symptoms at both time points.

**Table 6:** Phenotypic correlation, cross-twin cross-time correlations, genetic, shared and non-shared environmental correlations, and parameter estimates for hoarding symptoms in Swedish twins at age 15 and 18 years.

	Total sample	
Phenotypic correlation	0.40 (95% CI: 0.36–0.44)	
	**MZ**	**DZ**
Cross-twin cross-time correlations	0.25 (95% CI: 0.18–0.32)	0.15 (95% CI: 0.09–0.20)
**Model**	**ACE**	**AE**
*r*G	0.71 (0.39–1.00)	0.75 (0.57–0.94)
*r*C	1.0 (N.E.)	**-**
*r*E	0.21 (0.11–0.30)	0.20 (0.11–0.28)
Biv A	0.50 (0.15–0.85)	0.65 (0.50–0.79)
Biv C	0.12 (-0.11–0.36)	**-**
Biv E	0.38 (0.21–0.55)	0.35 (0.20–0.50)

**Note:** MZ = Monozygotic twins; DZ = Dizygotic twins; A = additive genetic effects; C = shared environmental effects; E = non-shared environmental effects and measurement error; *r*G = genetic correlation; *r*C = shared environmental correlation; *r*E = non-shared environmental correlation and measurement error; Biv A = proportion of the covariance between hoarding symptoms at age 15 and age 18 explained by additive genetic effects; Biv C = proportion of the phenotypic correlation between hoarding symptoms at age 15 and age 18 explained by shared environmental effects; Biv E proportion of the phenotypic correlation between hoarding symptoms at age 15 and age 18 explained by non-shared environmental effects and measurement error. N.E. = not estimable.

Parameter estimates for the best-fitting (AE) model along with the ACE model (for comparison) are shown in Table 6. According to the best-fitting model, we found a genetic correlation, *r*G = 0.75 (95% CI: 0.57–0.94) suggesting a strong genetic overlap in the covariance of hoarding symptoms at age 15 and age 18. The non-shared environmental correlation, *r*E = 0.21 (95% CI: 0.11–0.30), was considerably lower, but significant, indicating an overlap in non-shared environments that influence hoarding symptoms. However, since these correlations were less than unity, hoarding symptoms at both time points were also influenced by genetic and environmental factors whose effects were time-specific.

Finally, we estimated that 65% (95% CI: 50–0.79%) of the phenotypic correlation between hoarding symptoms at age 15 and at age 18 could be explained by additive genetic factors (i.e., bivariate heritability). The remaining covariance was explained by non-shared environmental effects (Biv E), 35% (95% CI: 20–50%).

## Discussion

This nationwide, population-based study examined the heritability of hoarding symptoms in the largest sample of young twins yet described in the literature. The results of this work have confirmed previous findings [7] regarding the heritability of hoarding in adolescence and extend these findings further throughout young adulthood. The contribution of genetic effects was substantial across the studied cohorts, though were somewhat lower than those observed in twin samples of older adults [10] and, furthermore, in contrast to our first hypothesis, appeared to slightly decrease over time from 41% in 15-year olds, to 31% in 18-year-olds and 29% in the oldest age groups. However, since confidence intervals of the heritability estimates were near-overlapping, cautious interpretation is warranted. In accordance with our second hypothesis, our longitudinal findings showed that hoarding symptoms were moderately stable (*r*Ph = 0.40) between ages 15 and 18. These results are in line with similar findings of the stability of obsessive-compulsive symptoms (OCS) during adolescence [18]. We also examined the sources of stability of hoarding symptoms between ages 15 and 18 and found that this was largely explained by genetic factors, while non-shared environmental factors largely had a time-specific effect, and contributed to stability to a lesser degree. The imperfect genetic correlation (*r*G) for hoarding symptoms between ages 15 and 18 is furthermore suggestive of possible different genetic effects on hoarding symptoms as age increases.

We also investigated the possibility of different genetic mechanisms operating in 15-year old boys and girls. Consistent with our previous study [7], which included a smaller subset of the current cohort our univariate analyses in this sample, suggested differences in genetic effects in boys and girls. While this finding underscores the potential for etiological sex-differences, model-fitting results suggest these differences are more consistent in the magnitude rather than source of genetic effects and may be confined to early adolescence. Moreover, keeping in mind that sex-differences were not reported in other young cohorts [15, 30], it cannot be ruled out that the observed sex-differences may not generalize to other cohorts. Clearly, further studies of the longitudinal development of hoarding symptoms throughout the lifespan are needed to understand the roles of age and gender, in the etiology of hoarding symptoms.

Within both sexes, and across age groups, non-shared environmental effects were found to account for a substantial portion of variance in hoarding symptoms. This contribution increased between follow-ups in the CATSS sample, ranging from 59% to 69% between CATSS-15 and CATSS-18, and was highest overall in the oldest sample (YATSS 20–28) at 71%. This finding could have clinical implications for the development of treatment strategies for hoarding symptoms in young people. Given the chronic and debilitating course of HD, and the treatment challenges associated with the disorder, a complementary clinical avenue to current treatment approaches would be to focus on preventing hoarding symptoms from deteriorating in to the full disorder. Indeed, an attempt at reducing hoarding symptoms in a non-clinical, sample of young adults was recently initialized [31]. Taking into account the role of non-shared environment suggested in our samples, refinement of such intervention and prevention strategies to address developmentally-relevant environmental factors may offer a pathway for improving outcomes among individuals at risk of developing HD.

The contribution of shared environment appeared negligible for all cohorts, with the exception of female twins in the youngest sample. Thus, using a considerably larger sample from the same cohort, the previous finding of shared environmental effects on 15-year old girls [4] was replicated in the current study. Given the scarcity of shared environmental effects in the twin literature, this finding is intriguing and raises the question of the sources of these effects. Although specific shared environmental factors that influence hoarding are yet to be identified, gender differences in key shared environmental factors have been found in studies of adolescent depression. Crawford [32] and colleagues, for instance, have suggested that parental distress and discord may be associated with internalizing symptoms among girls in mid-adolescence (mean age = 13.7), while no such relationship was observed for boys of a similar age. Similarly, in a study by Davies and Windle [33], maternal depressive symptoms were found to be associated with depressive symptoms only among adolescent girls. Although it is questionable whether these findings could be extended directly to hoarding symptoms, given the high comorbidity rates between HD and depression in adults [34, 35] and the notable co-occurrence of depression and hoarding symptoms in youth [36, 37] the potential for shared etiology is plausible. Moreover, consistent with recent findings suggesting that, for some individuals, HD might debut late in life [38], the large effects of shared environment in female twins could be indicative of two types of hoarding: an early-onset phenotype and a late-onset phenotype, each with different etiological mechanisms. A growing body of work has, for instance, noted deficits in executive functioning among older adults with HD that exceed those present in both healthy age-matched adults and young adults with clinically-significant hoarding difficulties [39, 40]. Given the findings observed here, it cannot be ruled out that such differing presentations reflect mechanistic variations rather than age-related disease progression. To what extent these or other factors are acting upon hoarding symptoms in adolescence, uniquely among girls, and at what age they come into effect, is therefore a question for further study. Certainly, the identification of such environmental influences could have meaningful etiological and clinical implications, suggesting for instance the value of complimenting individualized treatments with familial intervention approaches. Further studies would also benefit from including even younger twins in order to elucidate when possible non-shared environments first come to affect hoarding symptoms in girls.

Our results should be interpreted in light of several limitations. First, hoarding symptoms in the adolescent samples were captured using a measure, not previously validated for this age group. Consequently, the HRS-SR was slightly modified, to improve capture of clutter confined to the adolescent’s bedrooms, (due to the expectation that adolescents would have greater personal control over the state of this room than other areas of the shared home environment). Second, the version of the HRS-SR utilized in the young adult sample did not include an item evaluating impairment. This alteration, in particular, may have impacted the hoarding phenotype captured in this investigation, and by extension our prevalence and heritability estimates. However, because all items of the HRS-SR are heavily inter-correlated, we believe the impact of such modifications to be minimal.

## Conclusions

Hoarding symptoms are heritable from adolescence throughout young adulthood, although heritability appears to slightly decrease over time. Shared environmental effects were only found to contribute to hoarding symptoms in girls at age 15. The stability of hoarding symptoms between ages 15 and 18 is largely explained by genetic factors, while non-shared environmental factors primarily have a time-specific effect. The findings indicate that the importance of specific etiological factors might vary across sexes and the lifespan.
